# Disease-Specific Regions Outperform Whole-Brain Approaches in Identifying Progressive Supranuclear Palsy: A Multicentric MRI Study

**DOI:** 10.3389/fnins.2017.00100

**Published:** 2017-03-07

**Authors:** Karsten Mueller, Robert Jech, Cecilia Bonnet, Jaroslav Tintěra, Jaromir Hanuška, Harald E. Möller, Klaus Fassbender, Albert Ludolph, Jan Kassubek, Markus Otto, Evžen Růžička, Matthias L. Schroeter, The FTLDc Study Group

**Affiliations:** ^1^Max Planck Institute for Human Cognitive and Brain SciencesLeipzig, Germany; ^2^Department of Neurology and Center of Clinical Neuroscience, First Faculty of Medicine, Charles University and General University Hospital in PraguePrague, Czechia; ^3^Institute for Clinical and Experimental MedicinePrague, Czechia; ^4^Department of Neurology, Saarland University HomburgHomburg, Germany; ^5^Department of Neurology, University of UlmUlm, Germany; ^6^Clinic for Cognitive Neurology, University Hospital LeipzigLeipzig, Germany

**Keywords:** magnetic resonance imaging, progressive supranuclear palsy, atypical parkinsonism, support vector machine classification, voxel-based morphometry

## Abstract

To identify progressive supranuclear palsy (PSP), we combined voxel-based morphometry (VBM) and support vector machine (SVM) classification using disease-specific features in multicentric magnetic resonance imaging (MRI) data. Structural brain differences were investigated at four centers between 20 patients with PSP and 20 age-matched healthy controls with T1-weighted MRI at 3T. To pave the way for future application in personalized medicine, we applied SVM classification to identify PSP on an individual level besides group analyses based on VBM. We found a major decline in gray matter density in the brainstem, insula, and striatum, and also in frontomedian regions, which is in line with current literature. Moreover, SVM classification yielded high accuracy rates above 80% for disease identification in imaging data. Focusing analyses on disease-specific regions-of-interest (ROI) led to higher accuracy rates compared to a whole-brain approach. Using a polynomial kernel (instead of a linear kernel) led to an increased sensitivity and a higher specificity of disease detection. Our study supports the application of MRI for individual diagnosis of PSP, if combined with SVM approaches. We demonstrate that SVM classification provides high accuracy rates in multicentric data—a prerequisite for potential application in diagnostic routine.

## Introduction

Progressive supranuclear palsy (PSP) is a neurodegenerative disease with a clinical syndrome including atypical parkinsonism, supranuclear palsy, postural instability, and dementia. Neuropathologically, PSP is characterized by the accumulation of tau protein (tauopathy), resulting in neurofibrillary tangles and affecting both neurons and glial cells (Williams and Lees, 2009). It is associated with structural changes in the midbrain, also called “hummingbird” or “penguin sign”.

Recently, several studies investigated the pattern of PSP-related structural brain changes using magnetic resonance imaging (MRI) in combination with an analysis technique called voxel-based morphometry (VBM). These studies present, at least partly, conflicting results. Many studies found a diminished gray matter density (GMD) in the left and right insulae (Brenneis et al., 2004; Padovani et al., 2006; Ghosh et al., 2012). Several papers reported a reduced GMD in the thalamus (Cordato et al., 2005; Boxer et al., 2006; Padovani et al., 2006; Shi et al., 2013), but other papers did not find GMD changes here (Brenneis et al., 2004; Ghosh et al., 2012). Volumetric analysis of structural MRI showed a significantly smaller putamen in PSP patients in comparison to healthy controls (Messina et al., 2011). Recently, quantitative and systematic meta-analyses have been introduced to imaging data to identify the prototypical neural correlates of neurodegenerative diseases (Schroeter et al., 2007, 2008, 2009). Meanwhile three comprehensive meta-analyses have applied methods like anatomical likelihood estimates or effect-size signed differential mapping to PSP studies (Shi et al., 2013; Shao et al., 2014; Yu et al., 2015). By investigating gray matter changes, these meta-analyses consistently identified the thalamus, basal ganglia, insula, and midbrain as the disease-specific core network of PSP.

Our study aimed at further investigating structural brain changes associated with PSP by using MRI and VBM using a multicentric approach. We included a cohort of 20 patients and 20 healthy control subjects. Note that 20 patients is a relatively large cohort because of the low prevalence of PSP. We included 11 patients from Germany (Ulm, Homburg, Leipzig) and 9 patients from the Czech Republic (Prague). In order to assess the effect of the different locations and scanning conditions, we analyzed the Czech participants separately, because all Czech participants were scanned under identical conditions. In addition, we also performed a conjunction analysis using the Czech and the German cohort. A high degree of similarity between the VBM results of both cohorts would demonstrate that the effects of PSP in terms of brain degeneration are large enough to be shown across different centers using VBM.

Another aim of the study was the individual classification of PSP patients and healthy controls by machine learning pattern recognition algorithms applied to imaging data with our multi-centric approach. Here, we performed support vector machine (SVM) classification (Chang and Lin, 2011) on the basis of GMD images obtained with VBM. In two recent studies, this approach was already used to classify PSP patients from healthy controls within a unicentric setting (Focke et al., 2011; Salvatore et al., 2014). We demonstrate that high accuracy rates can also be obtained across different centers. Furthermore, the availability of multicentric data for the training of a classifier is a major advantage in the reliable detection of a rare condition. Recently, multicentric SVM classification was shown to achieve high accuracy rates for the differentiation between Alzheimer's disease and frontotemporal lobar degeneration (Dukart et al., 2013). Dukart and colleagues used disease-specific regions of interest (ROI) for SVM feature selection instead of using all brain regions. The ROIs were selected according to comprehensive meta-analyses. Similar to their approach (Dukart et al., 2013) and in contrast to other recent work (Focke et al., 2011; Salvatore et al., 2014), we used disease-specific ROIs based on meta-analytically extracted prototypical neural networks for PSP (Shi et al., 2013; Shao et al., 2014; Yu et al., 2015) and performed SVM classification using the voxels within these ROIs for feature selection in addition to whole-brain analyses. Note that the ROIs are defined here (Shi et al., 2013; Shao et al., 2014; Yu et al., 2015) in an independent cohort in a data-driven manner. We compared the resulting accuracy rates with the results from a whole-brain approach, expecting higher accuracy rates within the ROI-based technique. In addition, we validated the influence of different kernel functions and different approaches of feature selection on results.

## Methods

### Subjects

A cohort of 20 PSP patients (7 female, age 67.3 ± 7.8 years, mean ± standard deviation) was compared to a group of 20 age- and gender-matched healthy control subjects (8 female, age 66.3 ± 7.8 years). Multicentric data were obtained in Germany and the Czech Republic (see Table 1 for demographic details). The German cohort of 11 patients (4 female, age 69.0 ± 9.3 years) and 11 controls (4 female, 68.1 ± 7.8 years) was chosen from the data of the Consortium for Frontotemporal Lobar Degeneration at the centers of Ulm, Homburg, and Leipzig. The Czech sub-cohort of 9 patients (3 female, 65.2 ± 5.4 years) and 9 controls (4 female, 64.2 ± 7.6 years) was selected in Prague under identical conditions and was, therefore, additionally used to perform a unicentric analysis. Mean age and age variability did not differ between cohorts as investigated by two-tailed two-sample *t*-tests and *F*-tests for equal variances (all comparisons *p* > 0.2). The study was approved by the local ethics committees (Ethics Committee of the General University Hospital in Prague, Czech Republic; Ethics Committee of the University of Ulm, Germany; Ethics Committee of the University of Leipzig, Germany; Ethics Committee of the Saarland Medical Board, Homburg, Germany). All participants were carefully informed about the study and gave signed written consent in accordance with the Declaration of Helsinki.

**Table 1:** **Demographical and scanner data for patients and control subjects**.

**Patient ID**	**Age (years)**	**Sex**	**City**	**Scanner**	**Control ID**	**Age (years)**	**Sex**	**City**	**Scanner**
P08DE	55	m	Ulm	Allegra	C39DE	73	m	Ulm	Allegra
P42DE	74	f	Ulm	Allegra	C54DE	78	m	Ulm	Allegra
P40DE	61	f	Ulm	Allegra	C72DE	75	f	Ulm	Allegra
P48DE	65	m	Ulm	Allegra	C82DE	73	m	Ulm	Allegra
P50DE	64	m	Ulm	Allegra	C85DE	71	f	Ulm	Allegra
P72DE	60	m	Ulm	Allegra	C91DE	71	m	Ulm	Allegra
P55DE	79	f	Leipzig	Verio	C64DE	54	f	Leipzig	Verio
P79DE	70	m	Leipzig	Verio	C80DE	64	m	Leipzig	Verio
P15DE	85	m	Homburg	Skyra	C22DE	72	m	Leipzig	Verio
P32DE	67	f	Homburg	Skyra	C49DE	61	f	Leipzig	Verio
P92DE	79	m	Homburg	Skyra	C46DE	57	m	Leipzig	Verio
P01CZ	68	m	Prague	Trio	C04CZ	65	f	Prague	Trio
P08CZ	69	f	Prague	Trio	C06CZ	66	f	Prague	Trio
P12CZ	64	m	Prague	Trio	C07CZ	72	f	Prague	Trio
P15CZ	61	f	Prague	Trio	C09CZ	58	m	Prague	Trio
P18CZ	61	m	Prague	Trio	C19CZ	54	m	Prague	Trio
P21CZ	75	f	Prague	Trio	C25CZ	61	m	Prague	Trio
P26CZ	69	m	Prague	Trio	C29CZ	56	m	Prague	Trio
P32CZ	58	m	Prague	Trio	C31CZ	70	f	Prague	Trio
P33CZ	62	m	Prague	Trio	C34CZ	76	m	Prague	Trio

*CZ, Czech Republic; DE, Germany; f, female; m, male*.

### Data acquisition

T1-weighted structural brain images were acquired at all four centers using the magnetization-prepared rapid gradient-echo (MP-RAGE) sequence implemented on 3T MAGNETOM scanners (Siemens, Erlangen, Germany; Prague: MAGNETOM Trio; Ulm: MAGNETOM Allegra; Homburg: MAGNETOM Skyra; Leipzig: MAGNETOM Verio). All images were acquired with a nominal resolution of 1 × 1 × 1 mm^3^. Further imaging parameters are listed in Table 2. Note that the same acquisition parameters were used in Homburg and Leipzig, whereas a slightly different set of parameters was used at the other two sites, Prague and Ulm (longer echo time with a smaller imaging bandwidth per pixel).

**Table 2:** **Acquisition parameters of the MP-RAGE sequence at all four imaging centers**.

**Imaging center**	**Prague**	**Ulm**	**Leipzig**	**Homburg**
Scanner	Trio	Allegra	Verio	Skyra
Software	syngo MR B17	syngo MR A30	syngo MR B17	syngo MR D11
Flip angle	10°	8°	9°	9°
Repetition time(ms)	2,300	2,200	2,300	2,300
Echo time(ms)	4.43	4.38	2.98	2.96
Inversion time(ms)	900	1,200	900	900
Bandwidth(Hz/Px)	150	130	238	240
FoV	240 × 256	256 × 256	240 × 256	240 × 256
Image matrix	240 × 256 × 160	256 × 256 × 208	240 × 256 × 176	240 × 256 × 176
Nominal resolution(mm^3^)	1 × 1 × 1	1 × 1 × 1	1 × 1 × 1	1 × 1 × 1

*MP-RAGE, magnetization-prepared rapid gradient-echo; FoV, field of view; Px, pixel*.

### VBM analysis

Image processing was performed using the VBM 8 toolbox rev. 435 (Structural Brain Mapping Group, University of Jena, Department of Psychiatry, Germany) with Statistical Parametric Mapping 12 rev. 6,470 (The Wellcome Trust Centre for Neuroimaging, UCL, London, UK) and MATLAB 8.6 (R2015b, MathWorks, Inc, Natick, MA). GMD images were generated using the unified segmentation approach that presents a probabilistic framework combining image registration, tissue classification, and bias correction (Ashburner and Friston, 2005). Each voxel within the GMD images contains a measure of gray matter probability obtained by the unified segmentation approach. In order to account for volume changes during normalization, GMD was scaled by the amount of non-linear deformation that is also called modulation. To meet the assumptions of random field theory, GMD images were finally smoothed with a Gaussian kernel of 8-mm full-width at half-maximum (FWHM). Voxel-wise statistical analysis was performed with the general linear model implementing a two-sample *t*-test to compare PSP patients with healthy participants, controlling for age, sex, and total intracranial volume. Clusters were detected using a voxel-threshold of *p* < 0.001. To correct for multiple comparisons, a minimum cluster size of *k* > 1,000 was chosen to detect significant clusters with *p* < 0.05, family-wise error (FWE) corrected threshold on the cluster level (Nichols and Hayasaka, 2003).

To study effects induced by a single center, and to assess between-center variability arising from different location and hardware, statistical analyses were performed separately with patients and controls from Prague (unicenter approach) and with patients and controls from the German centers (multicenter approach). Due to the smaller numbers of patients in both subcohorts, a voxel-threshold of *p* < 0.005 was used. However, a minimum cluster size of *k* > 1,000 was again used to report significant clusters at *p* < 0.05, FWE-corrected. A conjunction analysis was performed including the Czech and the German cohort to investigate the overlap of the results between both groups of participants.

To test the variability between the German centers, a second conjunction analysis was performed using two cohorts generated by merging the participants from Prague and Ulm, and by merging the participants from Prague, Homburg, and Leipzig (Homburg and Leipzig used identical scanning parameters, see Table 2). In both cohorts, two-sample *t*-tests were performed to detect significant GMD differences between patients and controls using the same threshold as in the conjunction analysis described above (voxel-threshold of *p* < 0.005 in combination with a minimum cluster size of *k* > 1,000). Results of both analyses were combined using a conjunction analysis to investigate the overlap.

### SVM analysis

In order to differentiate PSP patients from healthy controls, SVM classification was performed with GMD images using the libSVM software package rev. 3.18 (Chang and Lin, 2011). The libSVM package offers open source software using the sequential minimization optimization algorithm (Platt, 1998) supporting SVM classification and regression. Classification accuracy was obtained by cross-validation using the “leave one out” approach by generating a set of 400 models, leaving a patient and a control subject out when building the classifier. Thereafter, it was checked if both remaining data sets were classified correctly. Sensitivity and specificity were computed from the number of correctly classified patients and controls. To assess the stability of classification results depending on kernel type and feature selection, the analysis was performed with two different kernels (linear, polynomial) and two different approaches of feature selection. First, voxels were used with SPM's gray matter tissue probability map using different minimum gray matter probabilities between 0 and 80% (all thresholds are listed in Table 3). Before thresholding, the gray matter tissue probability map was interpolated to meet the resolution of the GMD images obtained by using the VBM toolbox as described above. The gray matter tissue probability map was also smoothed with a Gaussian kernel of 8-mm FWHM, which is the same filter that was applied to the GMD images.

**Table 3:** **Accuracy, sensitivity, and specificity of support vector machine (SVM) classification with cross-validation generating 400 models excluding a patient and a healthy control when building the classifier**.

	**Linear kernel**						**Polynomial kernel**					
	**PSP**	**Ac (%)**	**HC**	**Ac (%)**	**Sens (%)**	**Spec (%)**	**PSP**	**Ac (%)**	**HC**	**Ac (%)**	**Sens (%)**	**Spec (%)**
0.8	**200**	50	**320**	80	71	62	**240**	60	**308**	77	72	66
0.7	**259**	65	**360**	90	87	72	**313**	78	**335**	83	83	79
0.6	**305**	76	**343**	86	84	78	**320**	80	**336**	84	83	81
0.5	**295**	74	**341**	85	83	76	**320**	80	**329**	82	82	80
0.4	**281**	70	**340**	85	82	74	**317**	79	**337**	84	83	80
0.3	**281**	70	**339**	85	82	74	**317**	79	**337**	84	83	80
0.2	**282**	70	**339**	85	82	74	**318**	80	**338**	84	84	80
0.1	**282**	70	**340**	85	82	74	**318**	80	**338**	84	84	80
0.001	**281**	70	**340**	85	82	74	**318**	80	**338**	84	84	80
M1	**299**	75	**360**	90	88	78	**337**	84	**345**	86	86	85
M2	**339**	85	**330**	82	83	84	**340**	85	**323**	81	82	84
M3	**337**	84	**342**	86	85	84	**340**	85	**294**	74	76	83
M4	**299**	75	**357**	89	87	78	**340**	85	**316**	79	80	84

*Columns with progressive supranuclear palsy (PSP) patients and healthy controls (HC) show the number of correctly classified participants for two different ways of generating models using a linear and a polynomial kernel. Feature selection was performed with SPM's gray matter tissue probability map using different thresholds, and with three different masks generated by the WFU Pickatlas (Masks: M1 = putamen+pallidum+midbrain; M2 = M1+caudate; M3 = M2+thalamus; M4 = M3+insulae).: Ac, Accuracy; Sens, Sensitivity; Spec, Specificity*.

The second approach of feature selection was based on disease-specific ROIs. ROIs were extracted from comprehensive meta-analyses on structural MRI changes in the gray matter in PSP from the literature (systematic literature search in PubMed on June 14, 2016, search terms: supranuclear palsy, meta-analysis, VBM). Based on three relevant meta-analyses (Shi et al., 2013; Shao et al., 2014; Yu et al., 2015), the disease-specific ROIs included the thalamus, basal ganglia, midbrain, and insula, because these brain regions were consistently impaired across all meta-analyses. Note that the definition of disease-specific ROIs in our study was based on comprehensive meta-analyses independent from our data and conducted across whole-brain studies only. ROIs were defined with the WFU-Pickatlas across the aforementioned brain regions. Finally, a model was generated from all patients and controls, and weights of voxels most relevant for SVM classification were detected using the libSVM package (Chang and Lin, 2011).

## Results

**Figure 1:**
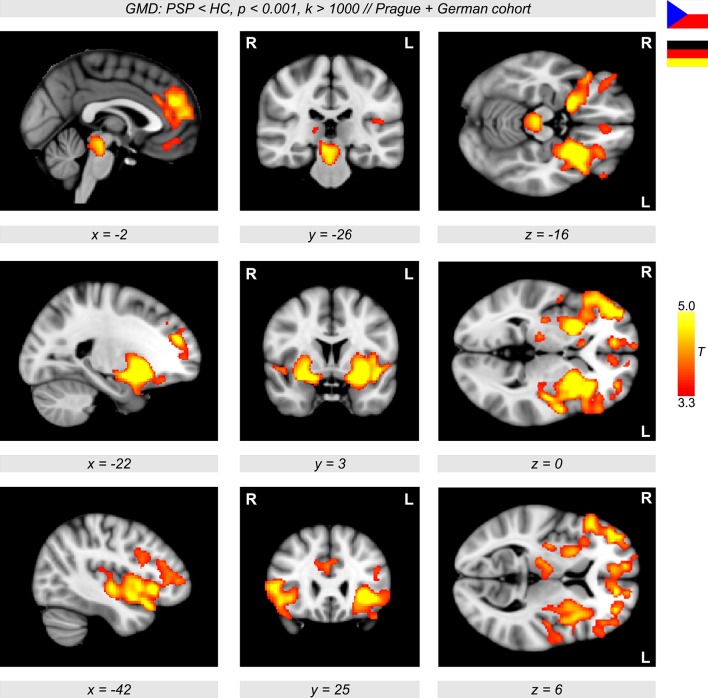
**Sagittal, coronal, and axial brain slices showing significant gray matter density (GMD) differences between 20 patients with progressive supranuclear palsy (PSP) and 20 age- and sex-matched healthy controls (HC) (color-coded in red/yellow, ***p*** < 0.001, ***k*** > 1,000, controlled for multiple comparisons using family-wise error-correction with ***p*** < 0.05 on the cluster level)**. In PSP patients, a diminished GMD was observed in the brain stem, insula, frontal cortex, and also in wide regions of the putamen extending to the pallidum.

**Figure 2:**
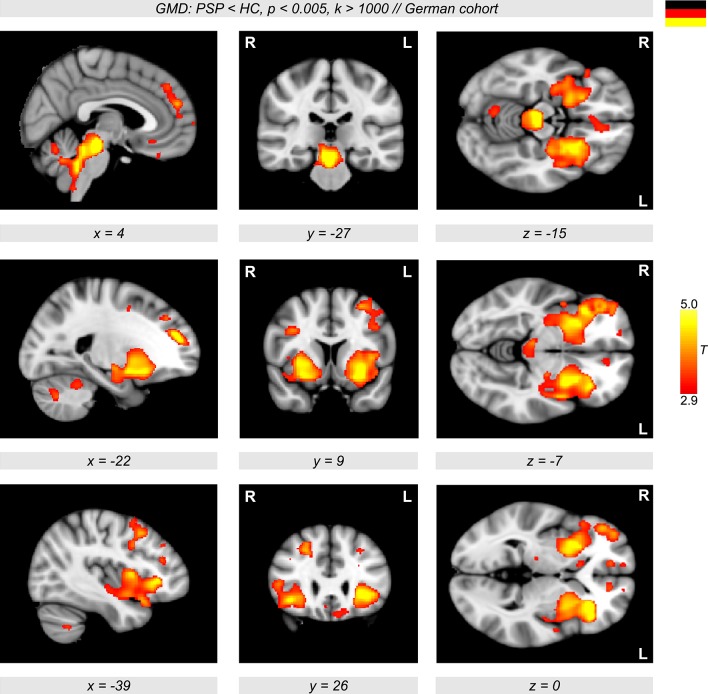
**Orthogonal brain sections showing significant gray matter density (GMD) differences between patients with progressive supranuclear palsy (PSP) and healthy controls (HC) (color-coded in red/yellow, ***p*** < 0.005, ***k*** > 1,000) in the German cohort**. Significant clusters are shown using family-wise error-correction with *p* < 0.05 on the cluster level.

The VBM analyses revealed a major decline in GMD in PSP patients compared to healthy controls. In particular, a diminished GMD was observed in the brainstem, thalamus, left and right anterior insulae, and also in wide regions in the putamen extending to the pallidum (Figure 1, color-coded in yellow/orange). Less prominent differences were detected in lateral orbitofrontal and frontomedian regions. The same regions were obtained when investigating GMD differences in the multicentric German cohort with patients and controls from three different centers (Ulm, Homburg, and Leipzig, Figure 2, color-coded in yellow/orange). The German PSP patients showed a diminished GMD in the brainstem, insula, and putamen/pallidum in both hemispheres. Smaller effects were also obtained in frontomedian regions.

**Figure 3:**
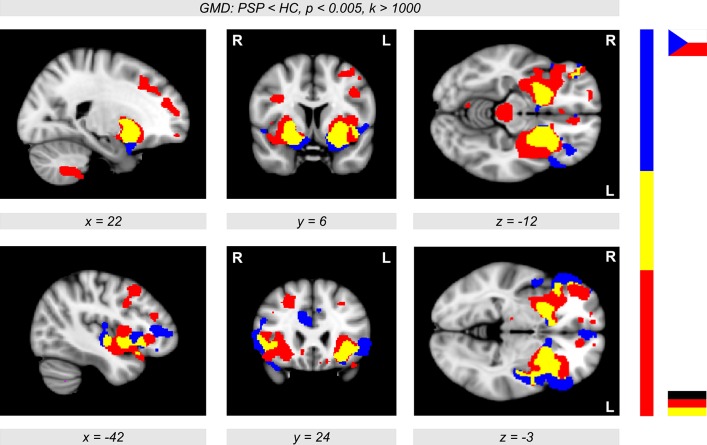
**Conjunction analysis showing both the unicentric Prague cohort (color-coded in blue) and the multicentric German sample (Ulm, Homburg, and Leipzig, color-coded in red, see also Figure 2) showing significant gray matter density (GMD) differences between patients with progressive supranuclear palsy (PSP) and healthy controls (HC) (***p*** < 0.005, ***k*** > 1,000)**. The overlap (color-coded in yellow) shows a reduced GMD in patients in the putamen extending to the pallidum, and also in the insular cortex. The German cohort showed prominent GMD reductions in the brainstem, while the Prague cohort also showed diminished GMD in frontomedian regions.

The conjunction analysis between the results obtained with the Prague cohort and German cohort revealed a consistent picture of GMD decline in PSP patients compared to controls. We obtained a remarkably large overlap of brain regions affected by PSP in both analyses with the German and Czech participants (Figure 3, colored in yellow). The overlap revealed a reduced GMD in patients not only in the putamen and pallidum but also in the insular cortex (colored in yellow). The German cohort showed prominent GMD reductions in the brainstem (colored in red), while the Prague cohort also showed diminished GMD in frontomedian regions (colored in blue).

**Figure 4:**
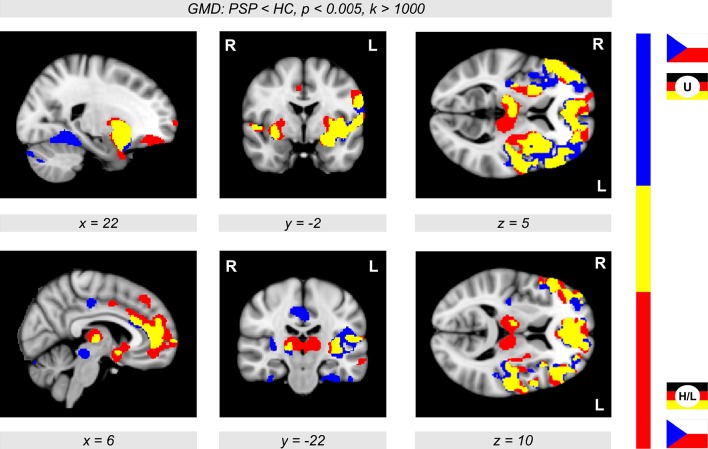
**Conjunction analysis showing significant gray matter density (GMD) differences (***p*** < 0.005, ***k*** > 1,000) in two groups of participants merging the participants from Prague and Ulm (U), and the participants from Prague, Homburg, and Leipzig (H/L)**. The overlap (color-coded in yellow) shows major reductions of GMD in patients with progressive supranuclear palsy (PSP) in the thalamus, putamen, insula, and also in frontomedian and frontolateral brain regions in comparison with healthy controls (HC).

Figure 4 shows the conjunction between PSP-related GMD decrease obtained from two different cohorts obtained by merging the participants from Prague and Ulm, and by merging the participants from Prague, Leipzig, and Homburg. An overlap of GMD decrease was observed in the thalamus, putamen, insula, and frontomedian and frontolateral regions (Figure 4, color-coded in yellow).

Using SVM classification with a polynomial kernel and a feature selection of voxels within SPM's gray matter mask with different minimum gray matter probabilities, PSP patients were identified with classification accuracy up to 80%. Healthy controls were detected with classification accuracy up to 84% (Table 3). The resulting sensitivity was up to 84% and the specificity was up to 81%. Interestingly, we received similar results using different gray matter probabilities ≤ 60% when choosing voxels within SPM's gray matter mask (Table 3). This demonstrates the robustness of SVM classification with respect to feature selection dependent on the minimum gray matter tissue probability. Using a linear kernel, accuracy values were generally lower for the PSP patients at around 70%.

**Figure 5:**
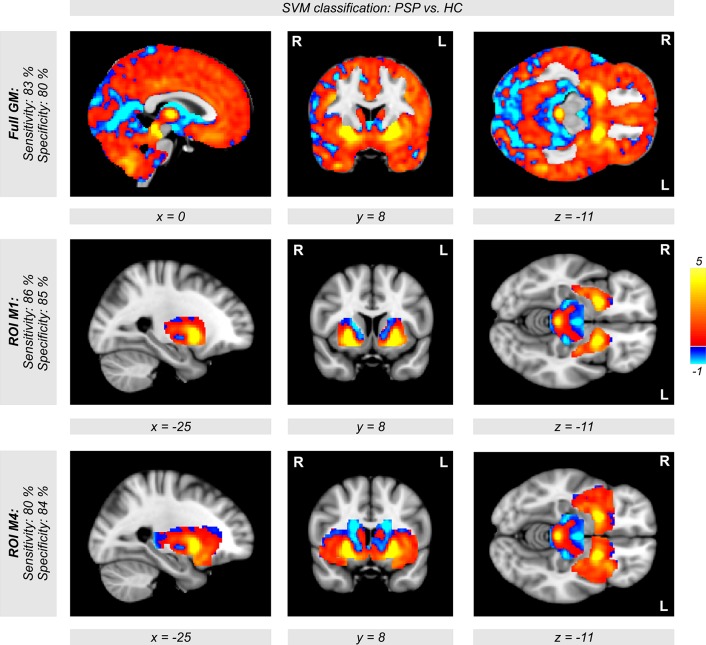
**Weights of voxels most relevant for support vector machine (SVM) classification between both groups of patients with progressive supranuclear palsy (PSP) and healthy controls (HC) after SVM training**. Note that these weights are relative and have no applicable units. Relevant voxels are located in the brain stem, putamen, and pallidum. Classification accuracy was obtained with a polynomial kernel using cross-validation generating a set of 400 models leaving a patient and a control subject out when building the classifier. SVM classification was performed on all voxels within the SPM's gray matter mask (tissue probability >0.4, top row). The middle and the bottom row show the same analysis using regions of interest instead of all gray matter voxels (middle row: M1 = putamen+pallidum+midbrain; bottom row: M4 = M1+caudate+thalamus+insulae).

The disease-specific ROI-based SVM approach focusing on the pallidum, putamen, caudate nucleus, thalamus, midbrain, and insula as PSP's core network generally outperformed the classification with all gray matter voxels of the brain with respect to disease detection and specificity of classification (Table 3, Figure 5). Using different ROIs and a polynomial kernel, we obtained accuracy rates above 84% for detecting PSP patients, which outperformed all other whole-brain approaches using minimum gray matter probabilities (see rows with masks M1–M4 in Table 3). Accuracies for detecting healthy control subjects varied between 74 and 86%, depending on the ROI. However, the specificity was always above 83%.

Finally, when using a model for all patients and healthy controls, relevant voxels for classification were observed in the brainstem, putamen, pallidum, and caudate nuclei, but also in cerebellar regions (Figure 5). Thus, using the whole-brain approach (first row in Figure 5), we observed the same regions that are discussed in the context of PSP in recent VBM studies (Shi et al., 2013; Shao et al., 2014; Yu et al., 2015).

## Discussion

Several recent studies aimed at investigating structural gray matter differences between PSP patients and controls using VBM (Price et al., 2004; Cordato et al., 2005; Ghosh et al., 2012). In line with these findings, we show a disease-related GMD decrease using a multicentric approach based on a relatively large sample of patients. A recent meta-analysis (Shi et al., 2013) investigating nine VBM studies with a total of 143 PSP patients suggested a crucial role of the insula and basal ganglia in PSP, which is corroborated by our findings. However, the most prominent finding (Shi et al., 2013) was a GMD decline in the thalamus, which we obtained when restricting the analysis to patients from Prague, Leipzig, and Homburg (see Figure 4, brain regions colored in red). Note that the thalamus is not included in the list of principal areas affected by the disease (Keith-Rokosh and Ang, 2008). Finally, we also found a major GMD reduction in gray matter regions in the vicinity of the brainstem, which suggests atrophy and seems to reflect the so-called hummingbird sign proposed as a pathognomonic entity in PSP.

In a more recent meta-analysis, Yu et al. (2015) studied 39 VBM publications investigating brain atrophy in PSP, corticobasal degeneration, and multisystem atrophy (MSA). In total, 176 PSP patients were included in this analysis. In line with our findings and the literature (Shi et al., 2013; Shao et al., 2014), they reported brain atrophy in insular brain regions and the thalamus. Our major result of GMD decrease in the putamen and pallidum appears divergent from Yu et al. (2015): While we excluded MSA patients from our cohort associating striatal GMD decline with PSP, Yu and colleagues (Yu et al., 2015) showed striatal GMD reduction solely for MSA, but not PSP. The authors claim that striatal atrophy might distinguish MSA from PSP (see Figure 2 in Yu et al., 2015). This is not in line with our findings, which show a more complex and widespread involvement of brain regions with PSP.

To distinguish different patterns of neurodegenerative diseases, SVM classification across GMD maps can be a useful tool beyond group comparisons. However, the question remains as to whether it is possible to achieve a robust dissociation between atypical Parkinson syndromes due to different patterns of brain degeneration. In a recent study, Focke et al. (2011) used SVM classification with a relatively small number of 10 PSP patients compared to 21 idiopathic Parkinson's disease (IPD) patients and 22 healthy controls. They did not observe a significant distinction between PSP patients and healthy controls based on GMD images (sensitivity 20%; accuracy 65.6%; see Table 4 in Focke et al., 2011). This is quite surprising considering the major GMD differences between PSP patients and controls that we observed. We achieved sensitivity, specificity, and accuracies above 80%. Relevant voxels for the SVM classification were located in exactly the same regions as detected with the VBM statistics investigating atrophy in PSP patients vs. controls. This major difference between our findings and the results of Focke et al. (2011) might be due to different disease stages or different sample sizes of the PSP patients. Probably, a sample of 10 PSP patients is a limitation for achieving sufficient sensitivity for classification. On the other hand, we also analyzed our German (11 patients) and Czech (9 patients) cohorts separately and received very similar GMD differences in both analyses (shown by the conjunction analysis in Figure 3). Unfortunately, Focke et al. (2011) did not show VBM comparisons between PSP patients and healthy controls, but only GMD differences between PSP and IPD; hence, whether the obtained differences are predominantly based on PSP or IPD cannot be disentangled. This within-disease comparison might be a reason why they (Focke et al., 2011) did not detect GMD changes in the striatum and insula, because of neurodegeneration in PSP and IPD in the same brain regions. Their cerebellar findings might be related to IPD and not to PSP. Note that both meta-analysis studies (Shi et al., 2013; Yu et al., 2015) do not report involvement of the cerebellum in PSP. It is even more interesting that Focke et al. (2011) obtained a significant SVM classification between PSP and IPD. However, they did not report a significant classification between patients and healthy controls— neither for PSP nor for IPD patients.

A more recent study used SVM classification with quite a large number of 28 PSP patients by performing a principal components analysis (PCA) on T1-weighted structural images (Salvatore et al., 2014). In contrast to the study of Focke et al. (2011), they also obtained high accuracy rates in comparisons of PSP or IPD patients with healthy controls with very similar patterns of relevant voxels for both conditions (Salvatore et al., 2014). Furthermore, they were able to dissociate PSP and IPD directly with relevant voxels mainly detected in the thalamus (Shi et al., 2013) and also in the cerebellum, which is in line with previous findings (Focke et al., 2011). In agreement with our results, Salvatore et al. (2014) detected relevant voxels in the medial part of the midbrain, whereas the striatum and insula did not contribute to their classification. This seems surprising because of the involvement of these regions in PSP as consistently shown in our study and previous meta-analyses (Shi et al., 2013; Yu et al., 2015). Whereas Salvatore et al. (2014) performed the SVM analysis using a PCA on T1-weighted images, we applied SVM to the GMD images. The combination of the SVM technique with the VBM approach might be more sensitive for relating gray matter changes in the striatum and insula to PSP.

Both previous SVM studies (Focke et al., 2011; Salvatore et al., 2014) applied a linear kernel for SVM classification, which reflects the default setting in the libSVM software package. However, other kernels might be more suitable, leading to higher accuracy rates and an improved sensitivity and specificity. In a recent study, Huppertz et al. (2016) performed SVM classification using a radial basis function (RBF) kernel. However, the extraction of SVM weighting factors is mathematically only defined for the linear kernel and not for an RBF kernel. Therefore, the SVM approach was repeated with a linear kernel to identify the most relevant regions for classification (Huppertz et al., 2016). Motivated by a recent paper demonstrating the advantage of using a polynomial kernel showing an improved accuracy when dissociating mild cognitive impairment from Alzheimer's disease (Belmokhtar and Benamrane, 2012), we also used a polynomial kernel for PSP disease classification. We clearly obtained an improved accuracy when comparing the accuracy rates with the use of a polynomial and a linear kernel. Note that the polynomial kernel allows kernel parameters that enable a change in the balance between specificity and sensitivity. In the future, this might be helpful when using sensitive approaches for disease detection.

In contrast to previous studies that used whole-brain approaches (Focke et al., 2011; Salvatore et al., 2014), we also showed that classification can be improved when using a disease-specific ROI-based approach for SVM feature selection. ROIs were defined in independent and comprehensive cohorts in a data-driven manner with meta-analyses across whole-brain studies (Shi et al., 2013; Shao et al., 2014; Yu et al., 2015) avoiding circular approaches. This is in line with previous work comparing the meta-analytically inspired ROI-based approach with the whole-brain approach for feature selection. Combining disease-specific ROI approaches with several imaging modalities can improve the classification accuracy that was shown for identification of and differentiation between Alzheimer's disease and frontotemporal lobar degeneration (Dukart et al., 2011, 2013). Therefore, future studies might combine ROI-based feature selection with additional imaging modalities, such as diffusion tensor imaging sensitive to changes in white matter or positron-emission tomography sensitive to metabolism, or clinical features and biomarkers from serum/cerebrospinal fluid for PSP detection that can help us to understand the specific pattern of disease-related brain atrophy in PSP. Further development of SVM-based classification might complement the radiologist's MRI-based diagnostics for PSP disease detection and characterization.

In sum, our study investigated structural brain differences between PSP patients and healthy controls. To pave the way for future application in personalized medicine, we applied SVM classification to identify PSP on an individual level. Using VBM, we found a major decline in GMD in the brainstem, insula, putamen, pallidum, and also frontomedian regions. SVM classification yielded high accuracy rates in multicentric data, a prerequisite for application in diagnostic routine. Focusing analyses on disease-specific ROIs and using an advantageous kernel led to higher accuracy rates. Our study supports the application of MRI for individual diagnosis of PSP, if combined with SVM approaches. Classification results might also be improved by advantageous kernel and feature selection.

## Author contributions

General conception: KM, RJ, CB, JT, JH, HM, ER, MS; Study design: KM, RJ, MS; Data analysis: KM; Figures: KM; Drafting the manuscript: KM; Final preparation of the article: KM, RJ, HM, MS; Members of the FTLD consortium: KF, AL, JK, MO, MS.

### Conflict of interest statement

The authors declare that the research was conducted in the absence of any commercial or financial relationships that could be construed as a potential conflict of interest.
